# Prevalence and risk factors of heavy menstrual bleeding in Africa: a narrative review

**DOI:** 10.1097/MS9.0000000000003394

**Published:** 2025-05-20

**Authors:** Emmanuel Ifeanyi Obeagu

**Affiliations:** Department of Biomedical and Laboratory Science, Africa University, Mutare, Zimbabwe

**Keywords:** Africa, epidemiology, heavy menstrual bleeding, reproductive health, risk factors

## Abstract

Heavy menstrual bleeding (HMB) is a common and impactful reproductive health issue that affects women worldwide, with significant implications for physical, emotional, and social well-being. In Africa, the prevalence of HMB is underreported, with estimates ranging from 10% to 30% in different regions. Factors such as limited access to healthcare, socioeconomic disparities, and cultural beliefs contribute to the high burden of HMB among African women. Despite the increasing recognition of its impact, substantial gaps remain in research and healthcare resources to address the condition comprehensively across the continent. Several risk factors contribute to the prevalence of HMB in African women, including chronic diseases like anemia, diabetes, and HIV, as well as reproductive health disorders such as fibroids and endometrial abnormalities. Nutritional deficiencies, particularly iron deficiency anemia, are widespread in many African populations, exacerbating the risk of excessive bleeding. Additionally, socio-cultural practices, including stigma and taboos surrounding menstruation, prevent timely diagnosis and treatment, further complicating management efforts.

**HIGHLIGHTS**

- **High Prevalence**: Heavy menstrual bleeding (HMB) affects a significant proportion of African women, often underreported due to cultural stigma and lack of healthcare access.
- **Iron Deficiency Anemia**: HMB contributes to widespread anemia, exacerbating maternal morbidity and poor quality of life.
- **Underlying Conditions**: Uterine fibroids, adenomyosis, and bleeding disorders are major risk factors, with genetic and environmental influences.
- **Healthcare Barriers**: Limited awareness, inadequate healthcare facilities, and financial constraints hinder timely diagnosis and treatment.
- **Socioeconomic Impact**: HMB affects productivity, school attendance, and mental health, reinforcing gender disparities in education and employment.

## Introduction

Heavy menstrual bleeding (HMB) is defined as excessive menstrual blood loss that interferes with a woman’s physical, emotional, and social well-being. It is a prevalent condition that affects women worldwide, yet its significance remains underappreciated in many parts of the world, particularly in Africa. The condition can range from mild to severe, with severe cases leading to anemia, fatigue, and a significant reduction in quality of life. Despite its impact, HMB is often underreported, misunderstood, or considered a normal part of the menstrual cycle, leading to delayed diagnosis and treatment, especially in regions with limited access to healthcare resources^[^1–3^]^. In Africa, data on the prevalence of HMB are scarce, with estimates varying widely across different regions and populations. A few studies suggest that up to 30% of African women experience some form of heavy menstrual bleeding, yet this number is likely an underrepresentation due to the lack of widespread screening, poor reporting mechanisms, and socio-cultural factors that discourage open discussion of menstrual health. The situation is further complicated by a significant lack of health education and awareness about menstrual health in many African communities, where women often do not seek medical help due to embarrassment or stigma associated with menstruation^[^4,5^]^.

There are multiple factors contributing to a higher prevalence of HMB in African women. One key determinant is socioeconomic status. In many African nations, women from low-income backgrounds are disproportionately affected by HMB, primarily due to inadequate healthcare access, poor sanitation, and nutritional deficiencies. Women in rural areas face additional barriers, such as long distances to healthcare facilities and limited availability of diagnostic services. In urban settings, while healthcare services may be more accessible, the burden of healthcare costs remains a significant obstacle for many women, limiting their ability to seek appropriate treatment^[^6–9^]^. Another prominent risk factor for HMB in Africa is the presence of chronic diseases such as anemia, diabetes, and hypertension. Anemia, particularly iron deficiency anemia, is highly prevalent in Africa due to nutritional deficiencies, parasitic infections, and blood loss from conditions like HMB itself. Studies have also linked HIV infection with menstrual irregularities, including HMB, as the disease can affect the immune system and alter hormonal regulation. Similarly, other reproductive health disorders such as fibroids and endometrial abnormalities have been identified as significant contributors to the occurrence of HMB in African women, with fibroids being particularly common in this population^[^10,11^]^. Cultural beliefs and practices surrounding menstruation also play a critical role in the prevalence and management of HMB. In many African cultures, menstruation is considered a private and sometimes taboo subject, leading to hesitancy in discussing menstrual problems openly. This cultural stigma can prevent women from seeking timely medical care, resulting in delays in diagnosis and treatment. Moreover, misconceptions about menstruation, such as the belief that heavy bleeding is a normal part of the menstrual cycle or a sign of fertility, can further discourage women from seeking help^[^12–15^]^. The lack of awareness and education regarding menstrual health is a key barrier to addressing HMB in Africa. Many women are unaware of what constitutes normal versus excessive bleeding and may therefore consider heavy menstrual bleeding as an inevitable part of womanhood. Health education on menstrual health, along with public health campaigns that address the importance of seeking medical care for abnormal menstrual bleeding, is essential in promoting better health outcomes. Without addressing this knowledge gap, the true extent of HMB and its associated complications will remain underappreciated, and many women will continue to suffer in silence^[^5,16,17^]^.

## Aim

The aim of this review article is to explore the prevalence and risk factors associated with heavy menstrual bleeding (HMB) in Africa, to assess the impact of HMB on women’s health, and to evaluate current management and intervention strategies.

## Review methods

The review process for this narrative was structured around a comprehensive search of the existing literature on heavy menstrual bleeding (HMB) in Africa. This approach involved several stages: defining the search parameters, selecting relevant studies, synthesizing data, and analyzing trends in prevalence, risk factors, impact, and management strategies.

**1. Literature Search Strategy**: The initial step involved an exhaustive search of academic databases, including PubMed, Google Scholar, and African Journals Online (AJOL). Studies published within the last two decades were prioritized to ensure that the data reflected the most current understanding of HMB in the African context. The search terms included combinations of keywords such as “heavy menstrual bleeding,” “prevalence,” “risk factors,” “Africa,” “management,” “intervention,” and “socio-cultural aspects.” In total, over 200 articles, reviews, and case studies were identified. However, only studies that provided data specific to Africa and addressed both the medical and socio-economic dimensions of HMB were included in the review.

**2. Study Inclusion Criteria**: The studies selected for inclusion were primarily those that focused on the prevalence and risk factors of HMB in various African countries, as well as those that discussed the impact of the condition on women’s health. Only articles published in peer-reviewed journals, with clear methodological descriptions and results, were considered. Studies that presented data on the effectiveness of management strategies, ranging from medical interventions to surgical procedures, were also included to provide a comprehensive overview of the available treatment options in the African context. Additionally, we prioritized research that addressed the role of cultural perceptions, healthcare infrastructure, and economic barriers in the management of HMB.

**3. Data Extraction and Synthesis**: Once the relevant studies were identified, data were extracted and organized into themes based on the objectives of the review. The prevalence of HMB was analyzed based on geographic regions, highlighting any variations between urban and rural areas or different African countries. Risk factors such as age, reproductive health history, socio-economic status, and the presence of conditions like uterine fibroids or polycystic ovary syndrome (PCOS) were examined. The impact of HMB on women’s physical and mental health, as well as its socio-economic implications, was also analyzed. Finally, management strategies, including pharmacological treatments, surgical interventions, and community-based approaches, were reviewed.

**4. Comparative Analysis**: To assess the diversity of experiences across Africa, a comparative analysis was conducted across different regions. The differences in healthcare access, availability of treatments, and cultural attitudes toward menstruation were considered. This allowed for an evaluation of how HMB is managed in settings with varying levels of healthcare infrastructure. Additionally, we looked at the effectiveness of existing interventions and identified gaps in care, particularly for women in low-resource settings.

**5. Narrative Synthesis and Reporting**: The findings from the selected studies were synthesized narratively, focusing on key themes such as the high prevalence of HMB, its significant impact on women’s health, and the challenges related to diagnosis and treatment. We also examined the role of public health education in reducing stigma and promoting better menstrual health practices. The synthesis was structured around a narrative form to provide a cohesive discussion that integrates the various facets of HMB, offering insights into the broader social, cultural, and medical context in Africa.

## Prevalence of heavy menstrual bleeding in Africa

The prevalence of heavy menstrual bleeding (HMB) in Africa is not well-documented due to the lack of comprehensive and large-scale studies. However, available data from smaller studies suggest that the condition is prevalent across various regions of the continent, affecting a significant proportion of women. The estimated prevalence of HMB ranges from 10% to 30% in different African populations. This variation in prevalence could be attributed to differences in study methodologies, geographic locations, and cultural factors. For example, a study in Nigeria reported that approximately 15% of women experienced symptoms consistent with HMB, while another study conducted in South Africa indicated a higher prevalence of 20%[18]. In addition to geographic variation, the prevalence of HMB is influenced by factors such as age, socioeconomic status, and access to healthcare. In urban areas, where healthcare services are more accessible, women may be more likely to seek medical advice and report symptoms of HMB. In contrast, women in rural areas may underreport the condition due to limited healthcare infrastructure, cultural taboos surrounding menstruation, and lack of awareness. As a result, the true prevalence of HMB in many African regions is likely underestimated[19]. Several risk factors contribute to the prevalence of HMB in African women. Chronic health conditions such as anemia, hypertension, and diabetes, which are prevalent in many African populations, have been linked to an increased risk of heavy bleeding. In particular, iron deficiency anemia is common in African women due to nutritional deficiencies, malaria, and blood loss from conditions like HMB itself. Furthermore, reproductive health disorders such as uterine fibroids and endometrial polyps are more common in African women, increasing the likelihood of HMB. Studies have shown that uterine fibroids are a significant contributor to HMB in African populations, with a higher prevalence of fibroids compared to women of other ethnic groups[20].

## Risk factors associated with heavy menstrual bleeding in Africa

The prevalence of heavy menstrual bleeding (HMB) in African women is influenced by a variety of risk factors, including both biological and socio-cultural determinants[21].

**1. Reproductive Health Disorders**: One of the most significant risk factors for HMB in African women is the presence of reproductive health disorders, particularly uterine fibroids. Fibroids, benign tumors in the uterine wall, are highly prevalent in African women and have been consistently linked to HMB. Studies have shown that African women have a higher incidence of fibroids compared to women of other ethnic groups, and this is thought to contribute to the higher rates of HMB observed on the continent. Endometrial polyps and adenomyosis, which are conditions where the tissue lining the uterus grows into the uterine muscle, are also prevalent and can cause excessive bleeding during menstruation. Additionally, conditions like pelvic inflammatory disease (PID) and endometriosis, though less frequently studied in Africa, may also contribute to the development of HMB^[^22,23^]^.

**2. Chronic Diseases**: Chronic diseases such as anemia, hypertension, and diabetes are prevalent in many African countries and significantly contribute to the risk of HMB. Anemia, particularly iron deficiency anemia, is widespread in sub-Saharan Africa due to poor nutrition, parasitic infections, and blood loss from other medical conditions like HMB itself. Women with anemia are more likely to experience excessive menstrual bleeding, as their bodies may have less capacity to compensate for blood loss. Similarly, diabetes and hypertension, both common in African women, have been linked to menstrual irregularities and can exacerbate the severity of HMB. These chronic conditions may affect hormonal regulation, vascular integrity, and blood clotting, all of which are important factors in menstrual health[22].

**3. Infectious Diseases**: HIV infection is another major risk factor associated with HMB in Africa. The continent bears a disproportionate burden of the global HIV epidemic, and HIV-infected women are more likely to experience menstrual disturbances, including HMB. HIV can directly affect menstrual function through its impact on the immune system and hormonal balance. Moreover, antiretroviral therapy (ART), used to treat HIV, can also cause menstrual irregularities in some women. Infections such as malaria, which is endemic in many parts of Africa, may also exacerbate the risk of HMB by contributing to anemia and other underlying conditions that affect menstrual health^[^24,25^]^.

**4. Nutritional Deficiencies**: Nutritional deficiencies, particularly iron, folate, and vitamin D deficiencies, are common in many African countries and have been associated with an increased risk of HMB. Poor diets, which are often deficient in essential nutrients due to poverty and limited access to healthy foods, contribute to the prevalence of iron deficiency anemia. Inadequate nutritional intake weakens the body’s ability to maintain blood volume and clotting, increasing susceptibility to excessive menstrual bleeding. Iron deficiency anemia, in particular, is a significant contributor to HMB, as the condition reduces the body’s ability to produce sufficient hemoglobin, thus exacerbating the bleeding during menstruation^[^26–28^]^.

**5. Socio-Cultural Factors and Healthcare Access**: Socio-cultural beliefs and practices related to menstruation also play a critical role in the risk factors for HMB. In many African communities, menstruation is often considered a taboo subject, leading to underreporting and a reluctance to seek medical advice. Stigma and shame associated with menstrual health may prevent women from seeking timely medical care, resulting in delayed diagnosis and inadequate management of HMB. Additionally, limited access to healthcare services, especially in rural and remote areas, hampers the ability of women to receive proper diagnosis and treatment. Inadequate healthcare infrastructure, coupled with high costs of medical care, prevents many women from addressing menstrual problems, including HMB, which may lead to the exacerbation of the condition over time^[^29–31^]^.

**6. Genetic and Environmental Factors**: Genetic predisposition and environmental factors can also contribute to the prevalence of HMB in Africa. Studies suggest that certain genetic variations may predispose women to conditions like fibroids, which are a known risk factor for HMB. Moreover, environmental factors such as exposure to endocrine-disrupting chemicals in the environment, pollution, and lifestyle changes may influence hormonal balance and increase the risk of developing HMB. However, research on the genetic and environmental factors contributing to HMB in African women remains limited and requires further investigation^[^32,33^]^.

**7. Age and Parity**: Age and reproductive history are key determinants of menstrual health in African women. Women in their reproductive years, particularly those in their 30s and 40s, are at an increased risk of developing HMB, as this age range is associated with higher rates of uterine fibroids and other reproductive health conditions. Additionally, women with higher parity, or those who have had multiple pregnancies, are at greater risk of developing HMB due to the cumulative effects of hormonal fluctuations and changes in uterine structure over time^[^34–39^]^.

## Impact of heavy menstrual bleeding

Heavy menstrual bleeding (HMB) can have profound effects on the physical, emotional, and social well-being of women, significantly impacting their quality of life. Although it is often underreported or misunderstood, the consequences of HMB can be far-reaching, especially in regions where healthcare access is limited and menstrual health is not openly discussed. In Africa, where HMB is common but frequently overlooked, its impact on women’s health is particularly significant^[^40,41^]^.

**1. Physical Health Consequences**: The most direct physical impact of HMB is the potential for severe anemia. Chronic heavy blood loss can lead to a reduction in hemoglobin levels, resulting in iron deficiency anemia. Symptoms of anemia, such as fatigue, weakness, dizziness, and shortness of breath, are common in women with HMB. If left untreated, anemia can exacerbate other health conditions and lead to a further decline in overall health, increasing the risk of complications during pregnancy or childbirth. Women suffering from HMB may also experience discomfort and pain, such as pelvic pain, cramps, and bloating, which can disrupt their daily activities and diminish their overall physical well-being. The chronic nature of bleeding can also lead to further complications, including infections and uterine fibroids, which can contribute to the persistence and worsening of symptoms^[^13,42,43^]^.

**2. Emotional and Psychological Impact**: The emotional and psychological toll of HMB can be severe, affecting women’s mental health and well-being. Many women with HMB experience feelings of embarrassment, frustration, and anxiety due to the constant fear of leakage, the disruption of daily activities, and the potential for stigmatization. The unpredictable nature of heavy bleeding can cause a loss of control, which can lead to increased stress and emotional distress. Over time, this can result in depression, anxiety, and a diminished sense of self-worth. The inability to discuss menstrual health openly in many African communities can compound these emotional burdens, as women may feel isolated and unsupported. The constant management of HMB, including the use of sanitary products and the disruption of social activities, can contribute to a loss of confidence and a reduced quality of life^[^44,45^]^.

**3. Social and Economic Consequences**: The social and economic impact of HMB is particularly evident in African countries where access to sanitary products and healthcare is limited. Women with HMB often face challenges in maintaining their social roles due to the physical and emotional burden of the condition. In some cases, women may miss work, school, or social gatherings due to the severity of their symptoms, leading to reduced productivity and potential economic hardship. The inability to afford or access adequate sanitary products may further contribute to social isolation, as women may be forced to stay home or miss important events. In some cases, the stigma associated with menstruation and HMB may prevent women from seeking help, exacerbating their condition and further limiting their social and economic opportunities. The economic burden of managing HMB can also be significant, as women may need to spend on medications, medical consultations, and sanitary products, which can be prohibitively expensive, especially in low-income settings^[^46–48^]^.

**4. Reproductive Health Impact**: HMB can also affect a woman’s reproductive health and fertility. In some cases, the underlying causes of HMB, such as fibroids, endometriosis, or polycystic ovary syndrome (PCOS), can impact fertility. The chronic blood loss associated with HMB may also affect the overall health of the reproductive system, leading to issues with conception or an increased risk of pregnancy complications. Women with untreated HMB may face challenges in managing their menstrual health and reproductive goals, including difficulties in family planning and a higher likelihood of miscarriage or other pregnancy-related issues. In African communities, where fertility is often highly valued, the inability to conceive or carry a pregnancy to term due to HMB-related complications can result in additional emotional distress and societal pressure^[^49–51^]^.

**5. Delayed Diagnosis and Treatment**: One of the major barriers to effectively managing HMB in Africa is the delay in diagnosis and treatment. The cultural taboo surrounding menstruation and the lack of awareness about what constitutes excessive bleeding can lead many women to suffer in silence. Many may not seek medical help, either because of shame, lack of education, or limited access to healthcare facilities. This delay in seeking help often leads to worsening symptoms and more severe complications, making treatment more challenging and costly. The absence of routine screening for menstrual disorders in many healthcare settings further contributes to the underdiagnosis of HMB. As a result, women often experience prolonged suffering without appropriate interventions^[^52,53^]^.

**6. Public Health Implications**: On a larger scale, the impact of HMB on women’s health can have significant public health implications. The widespread prevalence of HMB, coupled with its associated health consequences, contributes to the overall burden of disease in African countries. In areas where healthcare resources are already stretched thin, addressing menstrual health issues like HMB can take a backseat to more urgent health concerns. As a result, the broader public health outcomes, including maternal health and overall women’s well-being, may be compromised. Addressing HMB as part of reproductive and maternal health programs could have positive downstream effects, improving not only individual health outcomes but also the health of the community as a whole^[^44,54–56^]^.

## Management and intervention strategies for heavy menstrual bleeding in Africa

The management of heavy menstrual bleeding (HMB) requires a multifaceted approach, integrating medical, surgical, and supportive interventions to address both the physical and emotional aspects of the condition. In Africa, where resources may be limited, effective strategies must be tailored to the specific challenges faced by women in different regions, including access to healthcare, affordability, and cultural considerations. By combining evidence-based practices with culturally sensitive approaches, it is possible to significantly improve outcomes for women suffering from HMB^[^57–59^]^.

**1. Medical Management**: The first line of treatment for HMB often involves pharmacological interventions to control bleeding, address underlying causes, and prevent complications like anemia. Common medical treatments include the following:

- **Non-Steroidal Anti-Inflammatory Drugs (NSAIDs**): Drugs such as ibuprofen are often used to reduce menstrual blood loss and alleviate associated pain. These medications are widely accessible, inexpensive, and can be effective in managing mild to moderate cases of HMB^[^60,61^]^.
- **Hormonal Therapies**: Hormonal treatments, such as oral contraceptives, the levonorgestrel intrauterine system (IUS), and progesterone-only therapies, can regulate menstrual cycles and reduce menstrual bleeding. The use of hormonal interventions can be particularly beneficial for women with underlying conditions like uterine fibroids or endometriosis that contribute to heavy bleeding. However, hormonal treatments may not be appropriate for all women, particularly those with certain health conditions or preferences regarding fertility^[^62,63^]^.
- **Tranexamic Acid**: This antifibrinolytic agent helps reduce blood loss by inhibiting the breakdown of blood clots. It is especially useful in women who have a tendency to bleed excessively but may not want to use hormonal treatments. Tranexamic acid is often used in combination with other therapies to manage bleeding more effectively^[^63,64^]^.
- **Iron Supplementation**: For women suffering from anemia due to chronic blood loss, iron supplements are critical to replenish depleted iron stores. Along with iron, folic acid and vitamin B12 supplementation may be indicated to address deficiencies and improve overall blood health. It is important to regularly monitor iron levels and adjust supplementation as needed^[^65,66^]^.

**2. Surgical Interventions**: In cases where medical management fails or is not effective, or when there are underlying structural causes of HMB such as fibroids or endometrial polyps, surgical options may be considered. Surgical approaches can be tailored to the severity of the condition and the woman’s reproductive goals.

- **Dilation and Curettage (D&C**): This procedure involves scraping the lining of the uterus to remove any abnormal tissue. D&C can be effective in cases where polyps or other growths are causing bleeding but may not provide long-term relief for recurrent HMB^[^57,67^]^.
- **Endometrial Ablation**: This minimally invasive procedure involves the removal or destruction of the uterine lining to reduce bleeding. It is typically recommended for women who have completed their family planning and are not interested in preserving fertility. Endometrial ablation has proven effective in controlling heavy bleeding, but its success rate can vary depending on individual factors[57].
- **Myomectomy**: For women with uterine fibroids, a myomectomy, which involves the surgical removal of fibroids, can be an effective solution for reducing HMB. This procedure can be particularly beneficial for women who wish to preserve their fertility, as it removes the source of the excessive bleeding without affecting the uterus’s ability to carry a pregnancy[68].
- **Hysterectomy**: In severe cases, when all other treatment options fail, or when there are no plans for future pregnancies, a hysterectomy (removal of the uterus) may be considered. While this is a definitive solution to HMB, it is often a last resort due to its irreversible nature and the significant recovery period required[57].

**3. Supportive Care and Lifestyle Modifications**: In addition to medical and surgical interventions, supportive care and lifestyle modifications play an essential role in the management of HMB, particularly in settings where access to advanced medical care may be limited[69].

- **Nutritional Support**: Proper nutrition is crucial for managing the effects of HMB, particularly when anemia is present. A diet rich in iron, vitamins, and minerals, such as dark leafy greens, red meat, and fortified cereals, can help prevent and manage anemia. Additionally, nutritional counseling and the promotion of healthy eating habits should be part of public health efforts to address HMB.
- **Psychosocial Support**: Given the emotional and psychological impact of HMB, providing psychosocial support is vital for women who experience distress, anxiety, or depression due to their condition. Counseling services, support groups, and education about menstrual health can help women feel empowered and less isolated. Reducing the stigma associated with menstruation and heavy bleeding is an essential component of improving care for women with HMB, especially in African societies where menstrual health is often a taboo.
- **Access to Sanitary Products**: Ensuring access to affordable and quality sanitary products is critical to managing the physical discomfort and social stigma associated with HMB. In many parts of Africa, women struggle to access sanitary pads, which can exacerbate the impact of the condition. Providing low-cost or subsidized sanitary products and increasing awareness about menstrual health can improve the quality of life for women dealing with HMB.

**4. Addressing Cultural and Societal Barriers**: Cultural and societal beliefs often play a significant role in how menstrual health is perceived and managed. In many African communities, menstruation is seen as a private or taboo subject, which may lead women to avoid seeking treatment for HMB due to fear of judgment or embarrassment. Public health education campaigns that normalize discussions about menstruation and emphasize the importance of seeking medical care for excessive bleeding are essential to breaking these barriers. Involving community leaders and local influencers in these campaigns can also help dispel myths and encourage more women to seek care^[^70,71^]^.

**5. Strengthening Healthcare Systems**: Improving the overall healthcare infrastructure is essential for effective HMB management, particularly in rural or underserved areas. Ensuring that healthcare workers are trained to diagnose and manage HMB, along with the provision of necessary medical supplies and diagnostic tools, is fundamental. Additionally, improving access to primary healthcare and specialist services for women can facilitate earlier detection, better management, and improved long-term outcomes for women with HMB[6].

## Conclusion

Heavy menstrual bleeding (HMB) is a significant and often underrecognized health issue in Africa, with far-reaching consequences for women’s physical, emotional, and social well-being. The prevalence of HMB varies across the continent, influenced by factors such as cultural practices, healthcare access, and underlying health conditions. Despite the challenges faced in managing HMB in low-resource settings, effective management strategies, including pharmacological, surgical, and supportive care interventions, can significantly improve the quality of life for affected women. Medical treatments, such as NSAIDs, hormonal therapies, and antifibrinolytics, offer effective solutions for managing bleeding and preventing anemia. For more severe cases, surgical interventions, like endometrial ablation, myomectomy, and, in extreme cases, hysterectomy, provide lasting relief. However, addressing HMB requires more than just clinical intervention. Supportive care, such as nutritional support, psychosocial counseling, and improving access to sanitary products, is crucial for mitigating the wider impact of the condition on women’s lives. Equally important is the need for public health initiatives that address the cultural stigma surrounding menstruation, promote awareness, and encourage women to seek care.

## Data Availability

Not applicable as this is a narrative review.

## Research registration unique identifying number (UIN)

Not applicable as this is a narrative review.
